# Vaginal delivery in women with COVID-19: report of two cases

**DOI:** 10.1186/s12884-020-03281-4

**Published:** 2020-10-02

**Authors:** Dongmei Cao, Miaomiao Chen, Min Peng, Heng Yin, Guoqiang Sun

**Affiliations:** 1 Department of Obstetrics, Maternal and Child Health Hospital of Hubei Province, No. 745 Wuluo Road, Hongshan District, Wuhan City, 430070 Hubei Province China; 2 Department of Obstetrics, The First Affiliated Hospital of Chongqing Medical University, No. 1 Youyi Road, Yuzhong District, Chongqing, 400016 China

**Keywords:** COVID-19, Vaginal delivery, Intrauterine vertical transmission, Case report

## Abstract

**Background:**

During the ongoing global outbreak of COVID-19, pregnant women who are susceptible to COVID-19 should be highly concerned. The issue of vertical transmission and the possibility of neonatal infection is a major concern.

**Case presentation:**

**Case 1:** A 35-year-old pregnant woman with a gestational age of 37 weeks and 6 days was admitted to our hospital at the point of giving birth. Except for the abnormalities in her chest CT image, she was asymptomatic. She had an uncomplicated spontaneous vaginal delivery, and her infant was discharged home for isolation. Because of the positive result of the maternal swabs for SARS-CoV-2 obtained on the 2nd day after sampling, we transferred the mother to the designated hospital and followed up with her by telephone interviews. Luckily, it was confirmed on February 23 that the newborn did not develop any COVID-19 symptoms after observation for 14 days after birth.

**Case 2:** Another pregnant woman, with a gestational age of 38 weeks and 2 days, was also admitted to our hospital because of spontaneous labor with cervical dilation of 5 cm. Since she had the typical manifestations of COVID-19, including cough, lymphopenia, and abnormal chest CT images, she was highly suspected of having COVID-19. Based on the experience from case 1, we helped the mother deliver a healthy baby by vaginal delivery. On the 2nd day after delivery, the maternal nasopharyngeal swab result was positive, while the infant’s result was negative.

**Conclusion:**

There is still insufficient evidence supporting maternal-fetal vertical transmission for COVID-19-infected mothers in late pregnancy, and vaginal delivery may not increase the possibility of neonatal infection.

## Background

The 2019 novel betacoronavirus (2019-nCoV, also called SARS-CoV-2) disease, officially named COVID-19 by the World Health Organization (WHO), was identified in Wuhan, Hubei, China, in December 2019 [1, 2]. SARS-CoV-2 is the 3rd epidemic coronavirus after SARS-CoV and MERS-CoV [3]. As of April 15th, a total of 83,745 infected cases have been confirmed in China. The epidemic has been reported in at least 200 countries/territories/areas, with 1,862,778 cases outside of China. The WHO announced that COVID-19 was a Public Health Emergency of International Concern (PHEIC) on 30 January 2020. As pregnant women are susceptible to COVID-19 and some common obstetrical adverse events, such as preterm premature rupture of membranes (PPROM), preterm birth, fetal growth restriction (FGR), and neonatal death, are associated with maternal pneumonias [4–6], pregnant women should be highly concerned by obstetricians [7]. In this study, we reported the diagnosis and treatment process of two pregnant women infected with COVID-19 who had vaginally delivered live, full-term, singleton babies.

## Case presentation

### Case 1

**Fig. 1:**
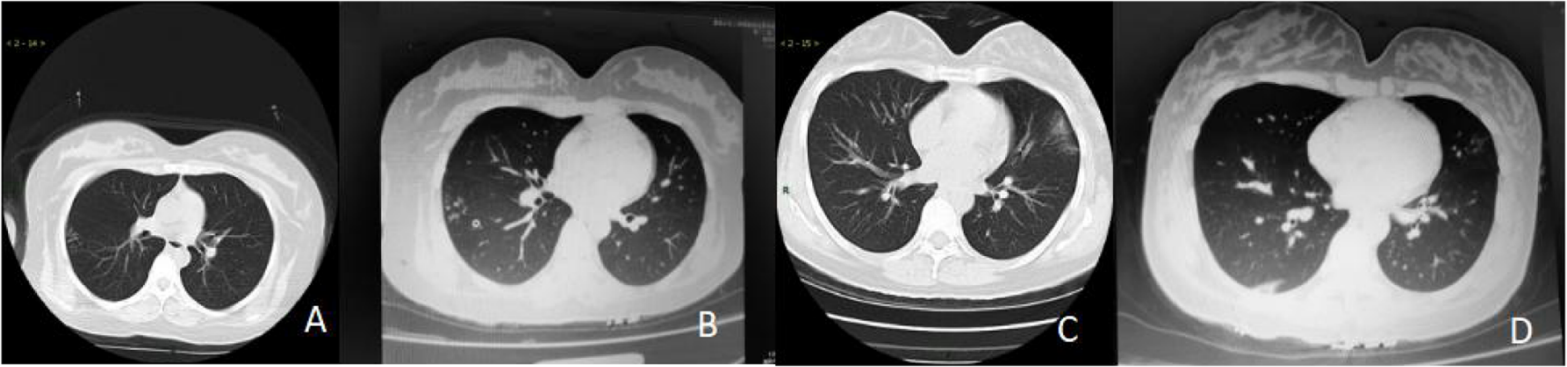
Transverse chest CT images. DA on February 8; B on February 13. **a** and **b** were from the first case: increased thickening of the lung texture, with spotted and slightly high-density shadows scattered on the lateral side of the right lower lobe. C on February 26; and D on February 29. C and D were from the second case: ground-glass opacity of the left upper lobe

A 35-year-old, primiparous woman in Wuhan city with a singleton pregnancy of 37 weeks and 6 days was admitted to our hospital (Maternal and Child Health Hospital of Hubei Province) due to regular uterine contractions at 17:50 on February 8, 2020, after undergoing chest CT scans. The chest CT images revealed spotted and slightly high-density shadows scattered on the lateral side of the right lower lobe (Fig. 1a). Except for lower abdominal pain, the pregnant woman had no signs or symptoms related to COVID-19 infection, and she reported normal fetal movement. None of her family members were diagnosed with COVID-19 or developed any respiratory symptoms. Since the woman developed cervical dilation of 0.5 cm and her CT result was abnormal, she was given a surgical mask instead of her original one. She was then transferred to a designated isolation delivery room by designated medical staff with specialized infection control preparation and strict infection protection equipment, including disposable work caps, medical protective masks, protective goggles, work clothes, disposable protective suit, disposable latex gloves, and disposable shoe covers.

The physical examination revealed a body temperature of 37 °C, pulse of 100 beats per minute, blood pressure of 135/86 mmHg, respiratory rate of 20 breaths per minute, and oxygen saturation of 99% while breathing ambient air. Considering that the pregnant woman and her family hoped that she would deliver vaginally and that there was no surgical indication for her, we decided to help her give birth naturally in the isolation delivery room. Fortunately, the labor went smoothly with no fever and no artificial intervention. The mother gave birth to a male infant with a birth weight of 3180 g at 04:58 on February 9, 2020. Apgar scores were 9 and 10 at 1 and 5 min, respectively. During her labor course, the laboratory tests (Table 1) showed significantly decreased lymphocyte counts (0.65 ×  10^9^/L), which is a common finding in patients with COVID-19 [8]. Then maternal nasopharyngeal swab samples were collected and sent to the designed hospital for testing for SARS-CoV-2 with the recommended kit in accordance with the WHO guidelines for qRT-PCR [9, 10]. The results were reported 2–3 days later. Considering the abnormalities in her chest CT images and lymphopenia on hematological examination, the mother was suspected of being infected with COVID-19; we asked the new parents for their opinions on breastfeeding and neonatal isolation, and they decided to temporarily isolate with the newborn and suspend breastfeeding. Then, the infant was discharged home for further isolation, and the mother was transferred to the designated isolation ward after 2 h of observation. Low-flow nasal oxygen inhalation, antiviral therapy and antibiotics were implemented for her.

**Table 1:** Laboratory results of the two mothers with COVID-19

	Case 1 Date				Case 2 Date		
	February 8 (before hospitalization)	February 9 (The day of delivery)	February 12	February 20	February 26 (before hospitalization)	February 26 (The day of delivery)	February 29
White blood cell count, × 10^9^/L	10.47	18.78↑	7.09	5.05	5.07	16.51↑	9.46
Neutrophil count, × 10^9^/L	8.55↑	17.08↑	5.41	2.93	3.94	15.67↑	7.5↑
Lymphocyte count, × 10^9^/L	1.33	0.65↓	1.19	1.56	0.92↓	0.60↓	1.62
Haemoglobin,g/L	125	109↓	98↓	123	121	121	113
Platelet count, ×10^9^/L	203	184	147	256	127	142	164
D-dimer, mg/L	2.0	10.36↑	/	/	1.69	8.31↑	1.56
Creatinine,mg/dL	5.61	11.98	/	/	7.92	0.110	/
Albumin, g/dL	38.3↓	34.4↓	33.4↓	39.3↓	41.1	34.0↓	35.6↓
AST, U/L	16.5	19.0	18	14	81.8↑	107.9↑	87↑
ALT, U/L	8.7	8.9	27	13	90.9↑	100.3↑	133↑
LDH, U/L	170.3	214	206	177	240	349.7↑	315↑
Influenza A and B	/	negative	/	/	/	negative	/
Parainfluenza	/	negative	/	/	/	negative	negative
Respiratory syncytial virus	/	negative	/	/	/	negative	negative
Adenovirus	/	negative	/	/	/	negative	negative
Chlamydia pneumoniae	/	negative	/	/	/	negative	negative
Mycoplasma pneumoniae	/	positive1:160	/	/	/	positive1:80	negative
SARS-CoV-2	/	positive	negative	negative	/	positive	negative

Note: *AST* Aspartate aminotransferase, *ALT* Alanine transaminase, *LDH* Lactate dehydrogenase

/ Not available

↑ The value in the patient was above the normal range

↓ The value in the patient was below the normal range

**Fig. 2:**
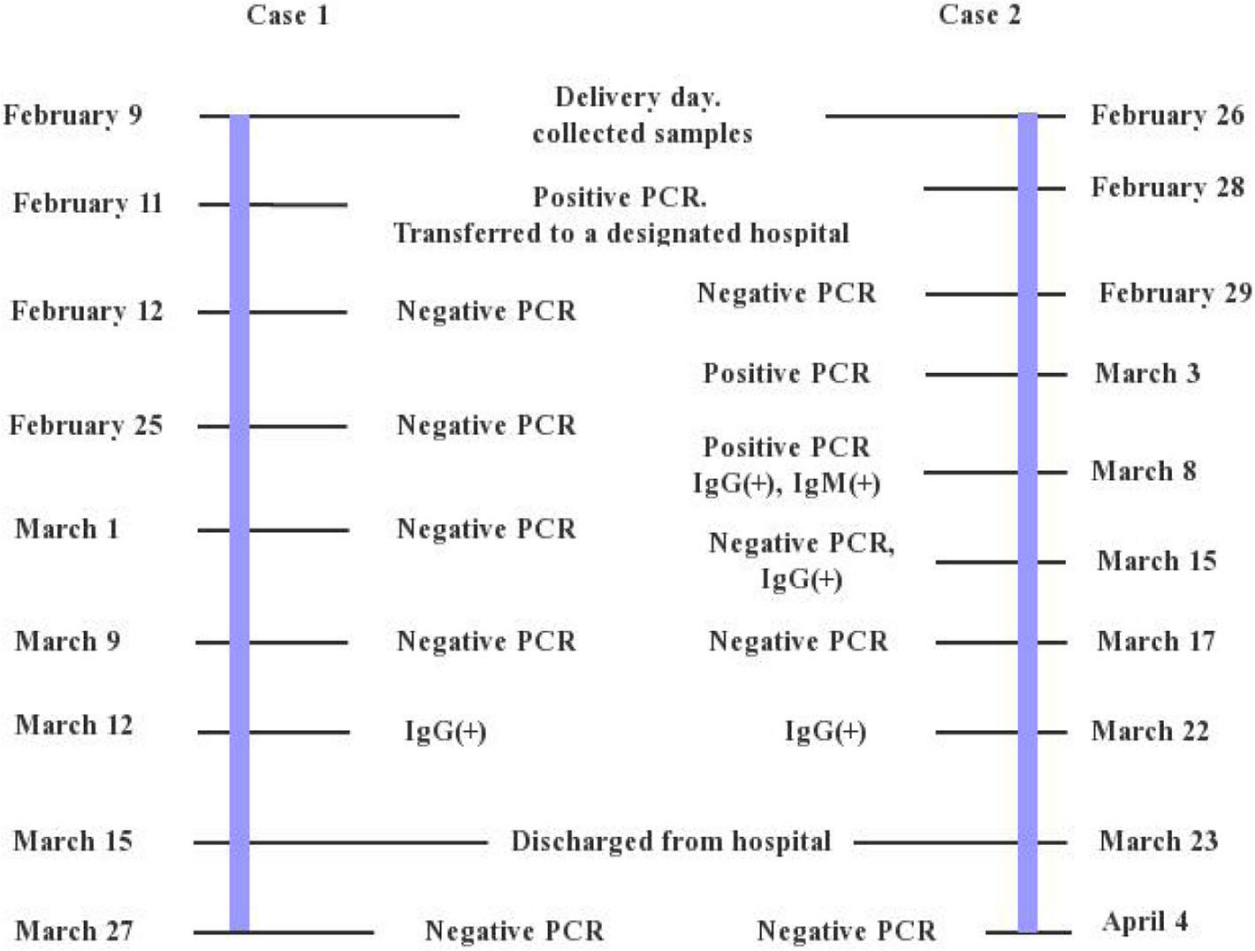
Time line of the patients’ nucleic acid test and serum antibody for SARS-Cov-2

The woman received a positive result for SARS-CoV-2 on the 2nd day after sampling (on February 11). According to this test result, we transferred the mother to the designated hospital and followed up with her by telephone interviews. Luckily, by February 23, which was the 14th day after birth, neither the newborn nor his father and grandmother who took care of him developed any COVID-19-related symptoms. The mother received antiviral (arbidol hydrochloride tablets), antibiotic (azithromycin dispersible tablets) and traditional Chinese medicine (Lianhua-Qingwen capsule) treatments after she was transferred to the designated hospital. During her hospitalization, she did not show any COVID-19-related clinical symptoms. The woman was tested five times, all of which were negative for SARS-CoV-2, after referral (Fig. 2). However, the immunoglobulin (Ig) G antibody for SARS-CoV-2 was detected in her serum sample on March 12, so she should be diagnosed as an asymptomatic carrier [11].

### Case 2

A 33-year-old pregnant woman (gravida 1, para 0) in Wuhan city, with a gestational age of 38 weeks and 2 days, was admitted to our labor and delivery unit for 6 h of lower abdominal pain at 10:20 on February 26, 2020, with a week-long history of dry cough and runny nose. However, she had not sought any medical care. Her transverse chest CT images revealed ground-glass opacities of the left upper lobe (Fig. 1c), and routine maternal outpatient blood tests showed lymphopenia (lymphocyte count: 0.92 × 10^9^/L, Table 1). This pregnant woman was admitted for spontaneous labor with a cervical dilatation of 5 cm, even though she was highly suspected of being infected with COVID-19. Similar to case 1, the woman was transferred to the designated isolation delivery room and was given a surgical mask to avoid droplets.

The physical examination revealed a body temperature of 36.5 °C, pulse of 99 beats per minute, blood pressure of 108/63 mmHg, and respiratory rate of 20 breaths per minute. With designated medical staff in attendance, the labor process progressed smoothly and resulted in the birth of a healthy female infant with a body weight of 2950 g at 11:28 on February 26, 2020. Apgar scores were 9 and 10 at 1 and 5 min, respectively. After being evaluated by a neonatologist, the newborn was transferred to a designated neonatal isolation ward immediately, and the mother was also transferred to another designated isolation ward after 2 h of postpartum observation. The mother also decided to temporarily suspend breastfeeding. Both the mother’s and her infant’s nasopharyngeal swabs for SARS-CoV-2 were collected (2 h after birth). The maternal laboratory tests (Table 1) showed elevated liver enzymes (AST: 107.9 U/L; ALT: 100.3 U/L) and decreased lymphocyte counts (0.60 × 10^9^/L). Reduced glutathione for injection was used to improve liver function, and the other treatments were the same as in case 1.

On February 28, we were informed that the maternal nasopharyngeal swab for SARS-CoV-2 was positive, and the infant’s swab was negative. Then the mother was transferred to the designated hospital and followed up via telephone. After she was transferred, antiviral, traditional Chinese medicine, and medicine for improving liver function were provided for her. Her symptoms gradually improved while she was in the designated hospital. The infant and the patient’s husband did not show any signs or symptoms related to COVID-19. Although the nucleic acid test for SARS-CoV-2 of the second patient was negative on February 29, the result was positive on March 3, and both SARS-CoV-2 IgG and IgM were positive on March 8. The patient should be confirmed with COVID-19.

Delayed cord clamping and skin-to-skin contact between the mother and infant were not permitted in either case. Because the two patients tested positive for SARS-CoV-2, the healthcare workers involved were temporarily quarantined for 14 days and tested after delivery. Luckily, none of the staff were infected during the deliveries. Further information on the two patients’ follow-up is provided in Fig. 2. The newborns in both two cases were followed for more than 14 days, and neither developed any COVID-19-related symptoms.

## Discussion and conclusion

Pathogen-host invasion stimulates the body’s immune response, and specific antibodies will be produced. Therefore, immunological techniques can be applied to achieve the rapid diagnosis of COVID-19 by detecting specific antibodies. Detection of serum IgM and IgG provides additional crucial evidence for the diagnosis of COVID-19 infection. For COVID-19, the production of IgG antibody occurs later than IgM production, while IgG antibody serum concentration is higher and those levels are maintained for longer than IgM. Therefore, a positive IgG antibody test probably indicates that the infection has developed to the middle stage or late stage [12]. Serum-specific antibodies such as IgM and IgG were included in the diagnostic criteria of COVID-19 on March 4 [11]. The detection of SARS-CoV-2 antibodies is of great significance for the diagnosis of suspected COVID-19 cases with a negative nucleic acid test (Additional file 1 [11]).

The first mother in our report was asymptomatic, and she did not show any clinical manifestations related to COVID-19 [13]. Although the infant’s nasopharyngeal swab was not collected in the first case, no signs of neonatal infection were reported during the telephone follow-up. The pregnant woman in case 2 had the typical manifestations of COVID-19, including cough, lymphopenia, and abnormal chest CT images, and her infant’s nasopharyngeal swab tested negative for SARS-CoV-2. As of April 10, neither the mothers nor fetuses in the two cases had poor obstetrical outcomes. The two cases in our study showed that there is still insufficient evidence supporting maternal-fetal vertical transmission of COVID-19 in late pregnancy, and there is no evidence that vaginal delivery would increase the possibility of neonatal infection. Our results are consistent with those of Chen HJ et al. [14] and Zheng QL et al. [15] Chen HJ et al. reported 9 pregnant women infected with COVID-19 in late pregnancy, but there was no obvious evidence supporting intrauterine infection via vertical transmission [14]. It is worth noting that all nine pregnant women underwent cesarean section with several obstetrical indications. In the study by Zheng QL et al., low expression of the SARS-CoV-2 receptor was detected in various cell types at the maternal-fetal interface by single-cell RNA sequencing on February 18, 2020 [15].

In the early stage of the outbreak of COVID-19, because of the limitation of the number of nucleic acid kits and the significance of chest CT images, many hospitals advised nonemergency pregnant women to undergo chest CT before being hospitalized. In these two cases, the abnormality of the CT images attracted our attention. Due to our timely isolation of the patients, the occurrence of hospital-acquired infection was avoided. Under the condition that a large number of patients cannot obtain the results of etiological examination in a timely manner, screening patients with transverse chest CT examination can achieve the purpose of “early detection and early isolation”. Previous data have shown that the lowest radiation exposure dose for adverse fetal outcomes is usually 50 ~ 200 mGy. The exposure dose of fetal radiation exposure from chest CT is 0.01 ~ 0.66 mGy [16]. There is no evidence that a single chest CT examination during pregnancy is harmful to the fetus. In clinical work, we must balance nucleic acid tests and transverse chest CT examinations to minimize under- and overdiagnosis.

In conclusion, there is still insufficient evidence supporting maternal-fetus vertical transmission of COVID-19 for pregnant women in late pregnancy, and vaginal delivery may not increase the possibility of neonatal infection.

## Supplementary information

**Additional file 1.** Interpretation of nucleic acid for SARS-CoV-2 combined with serum specific IgM and IgG antibodies.

## Data Availability

The datasets used during the current study are available from the corresponding author on reasonable request.

## Abbreviations

COVID-19: Coronavirus Disease 2019
SARS-CoV-2: Severe Acute Respiratory Syndrome Coronavirus 2
SARS-CoV: Severe acute respiratory syndrome coronavirus
MERS-CoV: Middle east respiratory syndrome coronavirus
qRT-PCR: Quantitative real time polymerase chain reaction
PPROM: Preterm premature rupture of membranes
FGR: Fetal growth restriction
WHO: World Health Organization
AST: Aspartate aminotransferase
ALT: Alanine transaminase
Ig: Immunoglobulin

## References

[1] WHO (2020). Novel coronavirus – China.

[2] Li Q, Guan X, Wu P (2020). Early Transmission Dynamics in Wuhan, China, of Novel Coronavirus-Infected Pneumonia. N Engl J Med.

[3] Corman VM, Muth D, Niemeyer D (2018). Hosts and sources of endemic human coronaviruses. Adv Virus Res.

[4] Lam CM, Wong SF, Leung TN (2004). A case-controlled study comparing clinical course and outcomes of pregnant and non-pregnant women with severe acute respiratory syndrome. BJOG.

[5] Wong SF, Chow KM, Leung TN (2004). Pregnancy and perinatal outcomes of women with severe acute respiratory syndrome. Am J Obstet Gynecol.

[6] Payne D, Ibrahim I, Sultan A (2014). Stillbirth during infection with Middle East respiratory syndrome coronavirus. J Infect Dis.

[7] Wang J, Qi H, Bao L (2020). A contingency plan for the management of the 2019 novel coronavirus outbreak in neonatal intensive care units. Lancet Child Adolesc Health.

[8] Wang D, Hu B, Hu C, et al. Clinical Characteristics of 138 Hospitalized Patients With 2019 Novel Coronavirus-Infected Pneumonia in Wuhan, China. JAMA. 2020. 10.1001/jama.2020.1585. 10.1001/jama.2020.1585 PMC7042881 32031570

[9] WHO (2020). Laboratory testing for 2019 novel coronavirus (2019-nCoV) in suspected human cases. Interim guidance.

[10] Tang A, Tong ZD, Wang HL, et al. Detection of Novel Coronavirus by RT-PCR in Stool Specimen from Asymptomatic Child, China. Emerg Infect Dis. 2020;26. 10.3201/eid2606.200301. 10.3201/eid2606.200301 PMC7258461 32150527

[11] National Health Commission of the People's Republic of China. Diagnosis and treatment of novel coronavirus infected pneumonia (trial 7th edition) [EB/OL].[2020⁃03⁃04]. http://www.nhc.gov.cn/xcs/zhengcwj/202003/46c9294a7dfe4cef80dc7f5912eb1989.shtml.

[12] Gao HX, Li YN, Xu ZG, et al. Detection of serum immunoglobulin M and immunoglobulin G antibodies in 2019-novel coronavirus infected cases from different stages. Chin Med J. 2020. 10.1097/CM9.0000000000000820. 10.1097/CM9.0000000000000820 PMC7339345 32221133

[13] Huang C, Wang Y, Li X (2020). Clinical features of patients infected with 2019 novel coronavirus in Wuhan, China. Lancet.

[14] Chen H, Guo J, Wang C (2020). Clinical characteristics and intrauterine vertical transmission potential of COVID-19 infection in nine pregnant women: a retrospective review of medical records. Lancet.

[15] Zheng QL, Duan T, Jin LP (2020). Single-cell RNA expression profiling of ACE2 and AXL in the human maternal–fetal interface. Reprod Dev Med.

[16] Committee on Obstetric Practice (2017). Committee Opinion No.723: Guidelines for diagnostic imaging during pregnancy and lactation. Obstet Gynecol.

