# Supplementary material for: Vaginal delivery in women with COVID-19: report of two cases
Source: BMC Pregnancy Childbirth. 2020 Oct 2;20:580. doi: 10.1186/s12884-020-03281-4 (PMC7530846; doi:10.1186/s12884-020-03281-4)
Supplement: Supplementary file 1 — Additional file 1. Interpretation of nucleic acid for SARS-CoV-2 combined with serum specific IgM and IgG antibodies. [file 12884_2020_3281_MOESM1_ESM.doc]

Appendix 1: Interpretation of nucleic acid for SARS-CoV-2 combined with serum specific IgM and IgG antibodies.

| Nucleic acid | IgM antibodies | IgG antibodies | Clinical significance |
| --- | --- | --- | --- |
| + | － | － | "Window phase" |
| + | + | － | Early stage of virus infection. |
| + | － | + | Middle- or late-stage of virus infection,  or recent secondary infection. |
| + | + | + | Active stage of virus infection. |
| － | + | － | Acute phase of virus infection. |
| － | － | + | Infected with SARS-CoV-2, but the virus in the body had been cleared. |
| － | Weak positive | － | Recent primary infection with very low viral load and the patient is on early stage. |
| － | + | + | Recently infected with SARS-CoV-2 and the patient is in the convalescent period, the virus is cleared; or the patient is in the active infection period, and the nucleic acid result is false negative. |

Note: +:positive; －:negative;

IgM: Immunoglobulin M; IgG: Immunoglobulin G.
